# OPTIMIZING PERIOPERATIVE CARE FOR PERIHILAR CHOLANGIOCARCINOMA: THE CRUCIAL ROLE OF MULTIDISCIPLINARY MANAGEMENT, NEOADJUVANT THERAPY, AND INTERVENTIONAL RADIOLOGY

**DOI:** 10.1590/0102-6720202400054e1848

**Published:** 2025-01-13

**Authors:** María Inés GAETE, José Donizeti de MEIRA, Soledad LOYOLA, Luís MENESES, Jorge DREYSE, Joaquín HEVIA, Eduardo BRICEÑO, Jorge MARTINEZ

**Affiliations:** 1 Pontificia Universidad Católica de Chile, Department of Digestive Surgery – Santiago, Chile.; 2 Universidade de São Paulo, Department of Gastroenterology – São Paulo (SP), Brazil.; 3 Pontificia Universidad Católica de Chile, Department of Radiology – Santiago, Chile.; 4 Clínica las Condes, Center for Critical Patients – Santiago, Chile.

**Keywords:** Cholangiocarcinoma, Postoperative complications, Hepatectomy, Pulmonary embolism, Nutrition therapy, Colangiocarcinoma, Complicações pós-operatórias, Hepatectomia, Embolia pulmonar, Terapia nutricional

## Abstract

**BACKGROUND:**

Perihilar cholangiocarcinoma presents unique challenges in perioperative management, requiring a comprehensive approach to optimize patient outcomes.

**AIMS:**

This case study focuses on the multidisciplinary management and innovative interventions performed in the perioperative care of a patient with hilar cholangiocarcinoma.

**METHODS:**

A comprehensive assessment and treatment strategy involving neoadjuvant therapy and interventional radiology techniques were implemented. Neoadjuvant chemotherapy was administered to reduce tumor size and improve resectability. The crucial role of interventional radiology in managing postoperative complications is highlighted, particularly in the case of massive pulmonary embolism.

**RESULTS:**

The neoadjuvant therapy successfully reduced tumor size, enabling an R0 surgical resection. Additionally, interventional radiology interventions, such as percutaneous pharmaco-mechanical thrombectomy, effectively addressed the life-threatening complication of massive pulmonary embolism.

**CONCLUSIONS:**

This article highlights the importance of a collaborative, multidisciplinary approach in managing complex oncological surgeries, especially regarding the hospital’s rescue capacity for severe postoperative complications. Emergent management with interventional radiology had a central role in resolving life-threatening complications.

## RESUMO

**RACIONAL:**

O colangiocarcinoma perihilar apresenta desafios únicos em seu manejo perioperatório, exigindo uma abordagem minuciosa para otimizar os resultados dos pacientes.

**OBJETIVOS:**

O manejo multidisciplinar e as intervenções inovadoras realizadas durante o cuidado perioperatório de um paciente com colangiocarcinoma hilar.

**MÉTODOS:**

Uma estratégia abrangente de avaliação e tratamento envolvendo terapia neoadjuvante e técnicas de radiologia intervencionista foram implementadas. Quimioterapia neoadjuvante foi administrada para reduzir o tamanho do tumor e melhorar a ressecabilidade. Destaca-se o papel crucial da radiologia intervencionista no manejo das complicações pós-operatórias, particularmente no caso de embolia pulmonar maciça.

**RESULTADOS:**

A terapia neoadjuvante reduziu com sucesso o tamanho do tumor, permitindo uma ressecção cirúrgica R0. Além disso, as intervenções radiológicas intervencionistas, como a trombectomia farmacomecânica percutânea, abordaram de forma muito eficaz a complicação potencialmente fatal da embolia pulmonar maciça.

**CONCLUSÕES:**

Este artigo destaca a importância de uma abordagem colaborativa e multidisciplinar no manejo de cirurgias oncológicas complexas, especialmente no que diz respeito à capacidade de resgate do hospital em complicações pós-operatórias graves. O tratamento emergencial multidisciplinar, com apoio da radiologia intervencionista, teve um papel central na resolução de complicações potencialmente fatais.

## INTRODUCTION

Cholangiocarcinoma (CCA) is a rare malignant neoplasm of the bile ducts in the Western world, although its incidence has increased progressively^10,11^. It is the second most common primary liver tumor after hepatocellular carcinoma, comprising 10–25% of cases^2^. Its incidence is higher in males and in the native American population, increasing with age, typically diagnosed in the seventh or eighth decade of life^2,7^. Arising from the bile duct epithelium and peribiliary glands, CCA generally has a discouraging prognosis, as most cases are diagnosed at advanced stages with published 5-year survival rates ranging from 10 to 50% and a median survival of 24 months^9^. Depending on anatomical location, CCA is classified as intrahepatic (iCCA), perihilar (pCCA), and distal CCA (dCCA); dCCA involves the common bile duct distally to the cystic duct’s insertion, pCCA the perihilar bile duct, between the insertion of the cystic duct and the second-order intrahepatic bile ducts, and iCCA the ducts proximal to the second-order branches of both the hepatic ducts and more proximal bile ducts. CCAs arising in the hepatic parenchyma are classified as iCCA^14^. Among diagnosed cases, pCCA, also known as Klatskin tumor, is the most common subtype, accounting for 60–70% of cases.

Surgical resection with negative microscopic margins (R0) is the only curative treatment, which is performed in 30% of CCA cases and 20–35% of pCCA cases, with high morbidity and significant perioperative mortality^3,13,16^. Liver transplantation, in selected cases, is another current option under discussion^4,6^.

Herein, we present the case of a patient with extensive pCCA treated in a multimodal approach, complicated by massive pulmonary embolism (PE), managed through coordinated multidisciplinary care, with extended survival for more than 9 years. The patient signed the informed consent to this report.

This case study focuses on the multidisciplinary management and innovative interventions performed in the perioperative care of a patient with hilar CCA.

## CASE REPORT

**Figure 1:**
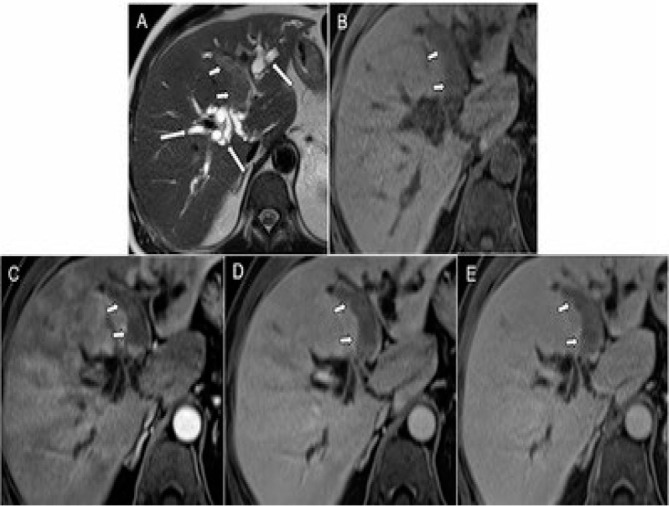
Axial MRI slices in axial T2SE sequence (A), T1GRE with pre-contrast fat saturation (B), and post-contrast in arterial phase (C), portal (D), and late 5 min (E) where an endoluminal mass (short white arrows) is observed hypointense on T1, with intermediate-high signal on T2, and hypovascular impregnation, which occupies and dilates the lumen of the medial segmental biliary branch. Note the dilation of the intrahepatic bile duct in the other segments (long white arrows).

**Figure 2:**
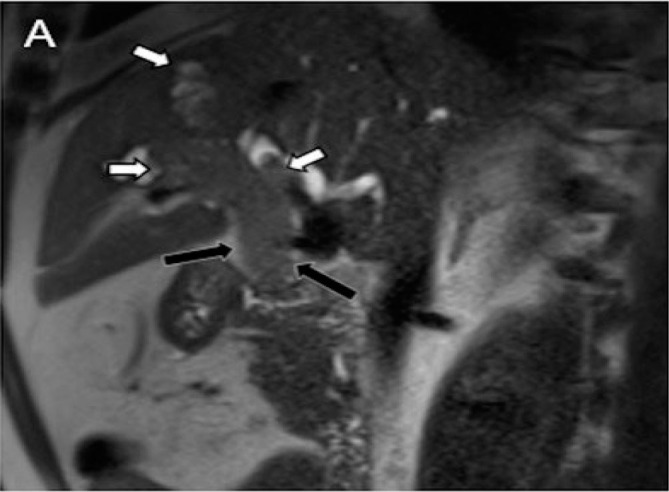
Sagittal T2SE acquisition showing intraductal mass with a component in the common hepatic duct (black arrows) and extension toward the medial segmental intrahepatic biliary branch (white arrows).

**Figure 3:**
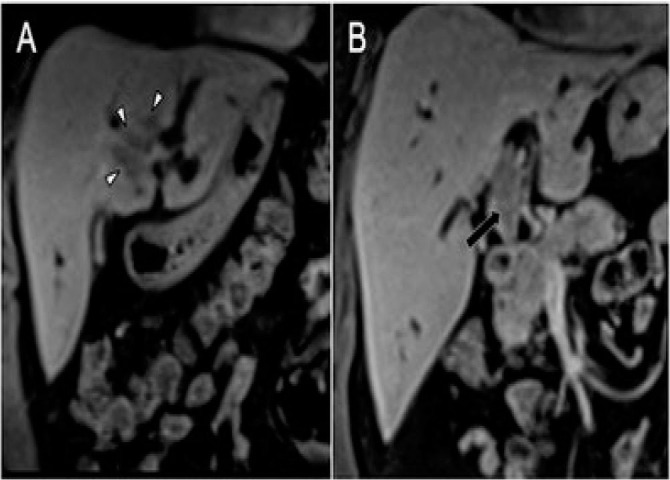
Post-contrast coronal T1 acquisitions showing luminal occupation of intrahepatic ductal branches by tumor mass (white arrowheads) (A) and the component that compromises the common hepatic duct (long black arrow) (B).

**Figure 4:**
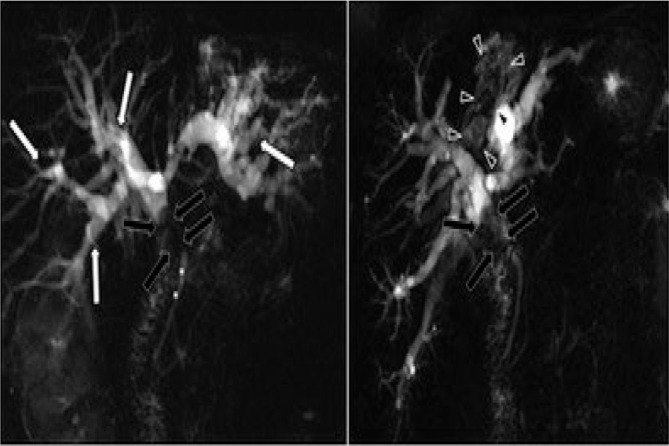
T2 cholangiographic sequences with thick slab technique showing absence of representation of the common hepatic duct due to luminal obliteration by the mass of the common hepatic duct (black arrows) (A), and representation of the tumor endoluminal component in the medial segmental intrahepatic biliary branch as an irregular endoluminal filling defect (black arrowheads). Note the marked dilation of the remaining intrahepatic bile duct (long white arrows) and the common bile duct of normal caliber and signal distal to the tumor obliteration of the common hepatic duct (asterisks) (B).

A 65-year-old male presented in April 2014 with a 2-month history of jaundice, dark urine, pale stools, pruritus, and weight loss of over 10 kg. Initial workup was significant for malnutrition and cholestasis (total serum bilirubin 19.3 mg/dL). A gadolinium-enhanced magnetic resonance imaging (MRI) showed an 11 mm intrahepatic biliary dilatation secondary to a 56 × 15 mm polypoid lesion in the intrahepatic bile duct of segment IV-A extending to the biliary confluence with intraluminal involvement of the common bile duct up to the head of the pancreas with left portal vein invasion and left liver lobe atrophy. There was no involvement of the confluence of the right secondary bile ducts (Figures 1–4). A contrast-enhanced computed tomography (CT) scan of the thorax, abdomen, and pelvis revealed no distant metastasis. Initially, it was approached as a stage III-B pCCA in a severely cachectic patient. Tumor markers Ca19-9, CEA, and CA-125 were within the normal range.

Our tumor board recommended performing percutaneous biliary drainage, cytology samples, nutritional support, and chemotherapy, with possible subsequent surgical resection based on treatment response.

**Figure 5:**
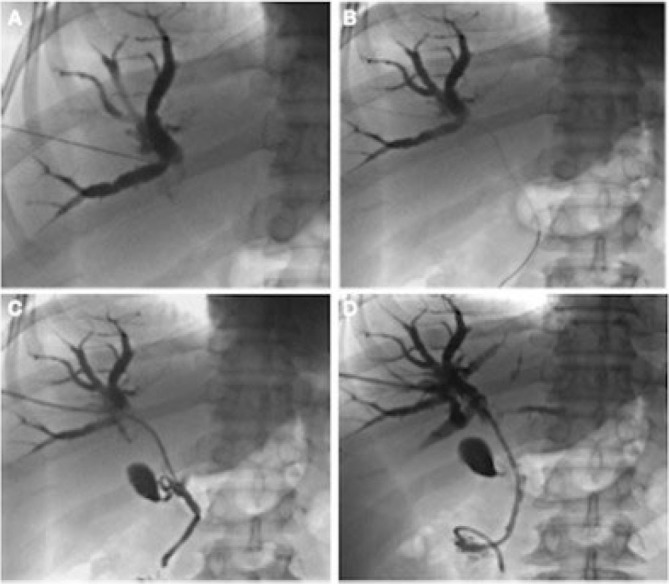
Percutaneous biliary drainage with endoscopic biliary prosthesis. (A) Right bile duct puncture. (B) Right bile duct cannulation. (C) Right bile duct dilation. (D) Drainage installation in the bile duct.

Percutaneous biliary drainage and cytology sampling were performed through the right hepatic lobe; a metallic stent (Figure 5) and a port-a-catheter were placed prior to neoadjuvant chemotherapy.

The patient completed six cycles of Gemcitabine and Cisplatin chemotherapy, achieving a significant tumor reduction to 48 × 13 mm on follow-up CT and MRI scans. Clinically, the patient regained 10 kg of weight and achieved complete resolution of jaundice.

### Surgical operation

Seven months later, the patient underwent exploratory laparoscopy, negative for peritoneal carcinomatosis. Subsequently, an open left hepatectomy was done, including an en-block caudate lobe and bile duct resection. The previous metallic stent was also removed during this procedure. Right bile duct and distal common bile duct frozen sections were negative for cancer. A concomitant lymphadenectomy of the hepatoduodenal ligament was performed. Biliary reconstruction was made with a Roux-en-Y hepaticojejunostomy to the confluence of the two right hepatic ducts.

### Postoperative course

**Figure 6:**
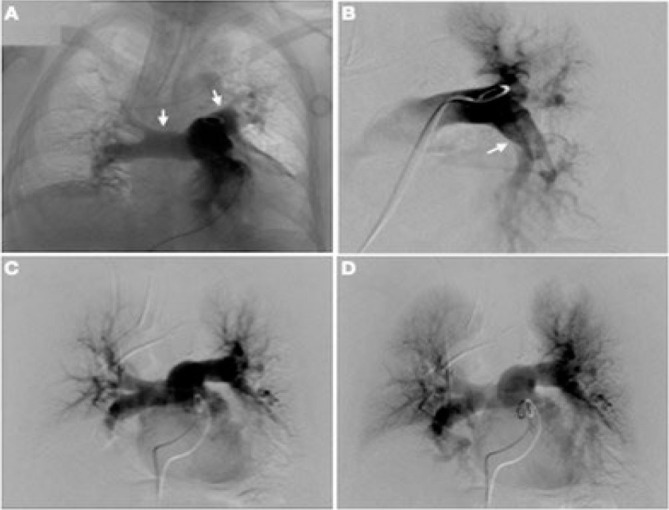
Pulmonary artery angiography. (A) Overriding thrombus in the trunk of the pulmonary artery. The arrows show how the pulmonary arteries are not contrasted. (B) Occlusion of the left inferior lobar artery. The arrow shows how the left inferior lobar artery is not contrasted. (C and D) Angiography after bilateral mechanical fragmentation and thrombolysis with local and intravenous r-TPA shows contrast in both pulmonary arteries.

Initially, the patient had a satisfactory postoperative recovery. Thromboprophylaxis was administered following our institutional protocol. On the third postoperative day, the patient suddenly developed dyspnea with peripheral oxygen saturation of 57%, hemodynamic instability, and peri-oral cyanosis requiring invasive mechanical ventilation and a vasopressor. An ultrasound was performed in the intensive care unit, revealing significant dilation of the right ventricle associated with impaired left ventricle contractility. A massive PE was diagnosed, and an urgent angiography was undertaken, showing a saddle embolus in the main pulmonary artery with occlusion of the left pulmonary lower lobar artery (Figures 6A and 6B). Systemic thrombolysis was contraindicated due to a recent major hepatectomy; then, he was treated with a percutaneous catheter and guidewire bilateral fragmentation with local administration of recombinant tissue plasminogen activator (r-TPA). Low-dose r-TPA intravenous thrombolysis was also administered (Figures 6C and 6D). A venous Doppler ultrasound of the lower extremities demonstrated bilateral infrapopliteal deep vein thrombosis (DVT), and an inferior vena cava filter was placed. Within 72 hours after these interventions, the patient had significant clinical improvement. The patient did not develop signs of liver insufficiency or bleeding.

The final pathology report was consistent with a semi-necrotic poorly differentiated infiltrating iCCA measuring 3.5 × 2.5 × 1.8 cm, mainly with intraluminal extension obstructing the left hepatic duct with negative surgical margins and no metastatic lymph nodes (AJCC 8th edition: pT1N0M0).

Over the next 5 years, close follow-up was done, with no evidence of recurrence and normal pulmonary function. Nine years after his oncological surgical intervention, he consented to report this publication.

## DISCUSSION

This clinical case demonstrates the challenges in the perioperative management of patients with hilar CCA and the potential development of serious postoperative complications, with reported morbidity and mortality rates ranging from 25.7 to 57.3% and 2.8 to 11.2%, respectively^3,13,14,16^.

Among the diverse postoperative complications, thromboembolic disease, including DVT and PE, particularly massive PE, pose a high risk of perioperative mortality with reported case fatality rates of up to 65%, depending on the degree of hemodynamic compromise and the timely initiation of effective management^1,5^. Our patient experienced a severe postoperative thromboembolic complication, specifically massive PE, with an absolute contraindication to one of the more common treatment alternatives of systemic thrombolysis due to a recent major hepatectomy. In cases where systemic thrombolysis is contraindicated, surgical embolectomy or pharmaco-mechanical catheter-directed therapy (PCDT) can be considered^1,12^. Our patient’s PCDT was chosen as an alternative, showing comparable outcomes to systemic thrombolysis when the latter is contraindicated^1,5,12^.

The role of neoadjuvant therapy for CCA, either with chemotherapy and/or radiotherapy, remains a topic of debate^8,15,17,18^. However, some authors suggest its use, especially in cases at high risk for not achieving R0 resection (borderline cases, such as our patient) or in cases of initially unresectable CCA, aiming to reduce tumor size and achieve conversion to resectability^8,18^. The use of neoadjuvant therapy is not associated with a significant increase in the rate of postoperative complications^15,17^. Wagner et al. demonstrated that the survival curve for cases requiring neoadjuvant therapy to achieve R0 resection was comparable to that of initially resectable patients^17^. Our patient’s neoadjuvant therapy reduced tumor size, enabling an R0 resection during surgery. Additionally, it provided time to improve the patient’s nutritional status before tumor resection.

## CONCLUSIONS

It is important to emphasize the critical role of a multidisciplinary approach in the perioperative management of an extended intrahepatic and hilar CCA. Neoadjuvant therapy was crucial in downstaging the tumor burden and improving resectability. Moreover, the pivotal role of interventional radiology in managing pre-operative workup and addressing severe postoperative complications is highlighted. Further research is warranted to evaluate the efficacy and implications of neoadjuvant therapy in CCA. At the same time, collaborative efforts between specialties are essential in optimizing outcomes for patients undergoing complex oncological surgeries.
